# Supplementary material for: Bayesian Linear Regression Modelling for Sperm Quality Parameters Using Age, Body Weight, Testicular Morphometry, and Combined Biometric Indices in Donkeys
Source: Animals (Basel). 2021 Jan 13;11(1):176. doi: 10.3390/ani11010176 (PMC7828516; doi:10.3390/ani11010176)
Supplement: Supplementary file 1 [file animals-11-00176-s001.pdf]

animals MDPI

Supplementary Materials: Bayesian Linear Regression Modelling for Sperm Quality Parameters Using Age, Body Weight, Testicular Morphometry, and Combined Biometric Indices in Donkeys

Ana Martins-Bessa [1,2,3,*], Miguel Quaresma [1,2,3], Belén Leiva [4], Ana Calado [1,2], and Francisco Javier Navas González [5]

1 Department of Veterinary Sciences, School of Agrarian and Veterinary Sciences, University of Trás-os-Montes and Alto Douro, Vila Real, Portugal; miguelq@utad.pt (M.Q.); anacalad@utad.pt (A.C.)
2 CECAV, Animal and Veterinary Research Center, University of Trás-os-Montes and Alto Douro, 5000-801 Vila Real, Portugal
3 Veterinary Teaching Hospital, University of Trás-os-Montes and Alto Douro, Quinta de Prados, 5000-801 Vila Real, Portugal
4 AEPGA- Association for the Study and Protection of Donkeys, Atenor, 5225-011 Miranda do Douro, Portugal; belenleiva.aepga@gmail.com
5 Genetics Department, Veterinary Sciences, University of Córdoba, Rabanales University Campus, Madrid-Cádiz Km. 396, 14014 Cordoba, Spain; fjng87@hotmail.com
* Correspondence: abessa@utad.pt; Tel.: +351-2593-50634

Table S1. Testing for normality using Shapiro–Francia W' test (for 50<n<2500 samples) for testicular biometry.

Variable	Observations	W'	V'	z	Prob>z
Age (months)	161	0.805	26.202	6.400	0.001
BW (Kg)	161	0.986	1.871	1.304	0.096
Ultrasound Length LT (cm)	161	0.908	12.383	5.015	0.001
Ultrasound Length RT (cm)	161	0.931	9.285	4.471	0.001
Ultrasound Height LT (cm)	161	0.971	3.885	2.778	0.003
Ultrasound Height RT (cm)	161	0.950	6.682	3.840	0.001
Ultrasound Width LT (cm)	161	0.931	9.262	4.466	0.001
Ultrasound Width RT (cm)	161	0.942	7.785	4.134	0.001
Ultrasound Volume LT (cm^3)	161	0.941	7.900	4.162	0.001
Ultrasound Volume RT (cm^3)	161	0.936	8.561	4.316	0.001
Ultrasound Total Testicular Volume (cm^3)	161	0.945	7.325	4.017	0.001
GSI (%)	161	0.957	5.701	3.531	0.001

W'—coefficient of concordance; V'—Pillay's trace; z—standard score.

Table S2. Testing for normality using Shapiro–Wilk test (for n<50 samples) for testicular biometry (n=16 testis) and spermatic data (n=40 ejaculates) of eight mature donkeys.

Variable	Shapiro-Wilk	df	Sig.
Weight	0.69	49	0.01
Length LT (cm)	0.78	49	0.01
Length RT (cm)	0.77	49	0.01
Height LT (cm)	0.79	49	0.01
Height RT (cm)	0.80	49	0.01
Width LT (cm)	0.76	49	0.01
Width RT (cm)	0.68	49	0.01
Volume LT (cm^3)	0.68	49	0.01
Volume RT (cm^3)	0.67	49	0.01
Total Testicular Volume (cm^3)	0.70	49	0.01
Gel-free volume (mL)	0.961	40	0.18
Sperm concentration ($\times 10^6$/mL)	0.965	40	0.24
TSN ($\times 10^9$)	0.881	40	0.01

Motility (%)	0.864	40	0.01
Morphologically normal (%)	0.856	40	0.01
Morphologically abnormal (%)	0.858	40	0.01
GSI (%)	0.914	40	0.01
TMS (× 10^9)	0.906	40	0.01

LT—left testicle; RT—right testicle; TSN—total sperm number; GSI—gonadosomatic index; TMS—total motile sperm; df—degrees of freedom.

Table 3. Bayesian Inference Pearson's Correlation Coefficient function output summary for US and caliper testicle biometry.

Items	Pearson correlation	Bayes factor	Mode	Mean	Variance	95% CI Lower Bound	95% CI Upper Bound
Length LT (cm)*Length LT (cm)	0.985	0.000	0.985	0.983	0.000	0.973	0.992
Length RT (cm)*Length RT (cm)	0.953	0.000	0.952	0.946	0.000	0.915	0.973
Height LT (cm)*Height LT (cm)	0.938	0.000	0.937	0.929	0.000	0.889	0.964
Height RT (cm)*Height RT (cm)	0.944	0.000	0.943	0.936	0.000	0.900	0.968
Width RT (cm)*Width RT (cm)	0.980	0.000	0.979	0.977	0.000	0.963	0.988
Width LT (cm)*Width LT (cm)	0.981	0.000	0.980	0.978	0.000	0.965	0.989
Volume LT (cm^3)* Volume LT (cm^3)	0.995	0.000	0.995	0.994	0.000	0.991	0.997
Volume RT (cm^3)* Volume RT (cm^3)	0.997	0.000	0.997	0.997	0.000	0.995	0.998
TTV (cm^3)*TTV (cm^3)	0.999	0.000	0.999	0.999	0.000	0.998	0.999
Ratio TTV/BW*Ratio TTV/BW	0.998	0.000	0.998	0.997	0.000	0.996	0.999

LT—left testicle; RT—right testicle; TTV—total testicular volume; BW—body weight.

Table S4. Commonly used thresholds to define significance of evidence through Bayes factor (BF).

Bayes Factor	Evidence Category	Bayes Factor	Evidence Category	Bayes Factor	Evidence Category
>100	Extreme Evidence for H_1	1-3	Anecdotal Evidence for H_1	1/30-1/10	Strong Evidence for H_0
30-100	Very Strong Evidence for H_1	1	No Evidence	1/100-1/30	Very Strong Evidence for H_0
10-30	Strong Evidence for H_1	1/3-1	Anecdotal Evidence for H_0	1/100	Extreme Evidence for H_0
3-10	Moderate Evidence for H_1	1/10-1/3	Moderate Evidence for H_0		

H_0: Null Hypothesis/Factors in the model do not have an effect over dependent variables/model comprising factors and the intercept and other only comprising the intercept present the same likelihood to predict for dependent variables; H_1: Alternative Hypothesis/Factors in the Model have an effect on dependent variables/model comprising factors and the intercept presents a higher likelihood to predict for dependent variables than one just comprising the intercept. Accessed from Jeffreys (1961) and Lee and Wagenmakers (2013).

Table S5. Model validity and accuracy parameters definition and interpretation.

Model criteria	Parameter	Definition	Interpretation	Reference
Model Validity	Acceptance rate (AR)	The acceptance rate is the proportion of proposed values of θ that were included in our final Markov chain Monte Carlo (MCMC) sample.	The asymptotically optimal acceptance rate is 0.234 under quite general conditions	Roberts, Gelman, and Gilks (1997).
	Efficiency (Ef)	Efficiency describes mixing properties of the Markov chain.	High efficiency means good mixing (low autocorrelation) in the MCMC sample, and low efficiency means bad mixing (high autocorrelation) in the MCMC sample. An efficient MH sampler has an AR between 15% and 50% and low autocorrelation and thus relatively large effective sample size (ESS) for all model parameters.	Roberts and Rosenthal (2001)
	Monte Carlo standard error (MCSE)	The Monte Carlo standard error (MCSE) is an approximation of the error in estimating the true posterior mean.	The lowest the MCSE the most accurate is the prediction of true posterior mean	Flegal (2008)
Model Accuracy	The posterior predictive *P* values provides (PPP values)	Computed as a goodness of fit measure for the model being tested. PPP values is the proportion of time during an MCMC run that a chosen test statistic, generated from a distribution predicted by the model, is higher than the test statistic generated from the distribution of the actual input data.	A value around 0.5 indicating a plausible good-fitting model and values toward the extremes of 0 or 1 indicating that the model is not plausible.	Defined in Appendix C of Lee and Song (2003)
	The marginal likelihood, also known as the evidence (Ev)	Denominator of the Bayes equation. The Log marginal likelihood is the combination of a data fit term and complexity penalty.	The model reporting the highest log marginal likelihood is precisely the model that is the best sequential predictor of the data tested according to the log scoring rule. A difference of 0.01 between two log-likelihood values is considered to be the same model. A difference of more than 3 log likelihood units (considered as "strong evidence against competing model" can be used as threshold for accepting a more parameter-rich model. A positive log likelihood means that the likelihood is larger than 1. This is possible because the log likelihood is not itself the probability of observing the data, but just proportional to it.	Chickering and Heckerman (1996), Aydin, Marcussen, Ertekin, and Oxelman (2014) and Edwards (1992).

Table S6. Descriptive statistic for US testicular in six juveniles and 17 matures donkeys (n=46 testis) and caliper measurements after orchiectomy in seven of these donkeys (n=14 testis).

Items	Juvenile (n=6)								Mature (n=17)							
	Mean	Std. Error of Mean	Std. Deviation	Minimum	Maximum	Percentile 25	Median (Percentile 50)	Percentile 75	Mean	Std. Error of Mean	Std. Deviation	Minimum	Maximum	Percentile 25	Median (Percentile 50)	Percentile 75
Lenght LT (cm)	3.31	0.06	0.37	2.80	3.87	2.92	3.39	3.50	8.22	0.11	1.24	5.51	10.60	7.43	8.20	9.08
Lenght RT (cm)	3.10	0.09	0.59	2.36	4.10	2.50	3.10	3.41	8.12	0.12	1.34	5.51	10.10	7.20	8.02	9.37
Height LT (cm)	2.04	0.06	0.38	1.50	2.63	1.77	2.02	2.30	4.90	0.10	1.06	3.24	6.93	3.82	4.85	5.51
Height RT (cm)	2.00	0.07	0.43	1.40	2.56	1.62	1.96	2.50	4.65	0.09	1.02	2.92	7.61	4.08	4.62	4.97
Width LT (cm)	2.36	0.09	0.57	1.50	3.37	2.00	2.39	2.50	6.17	0.11	1.18	3.32	7.88	5.40	6.17	6.92
Width RT (cm)	2.45	0.09	0.58	1.60	3.51	2.10	2.48	2.55	6.13	0.12	1.26	3.07	8.40	5.56	6.35	6.94
Volume LT (cm^3)	9.09	0.71	4.63	3.30	17.95	6.25	8.07	10.94	141.01	6.35	69.32	41.20	283.91	81.32	139.58	177.07
Volume RT (cm^3)	8.79	0.82	5.32	2.93	19.28	5.10	7.49	10.47	130.68	6.00	65.49	30.87	297.64	87.77	121.27	166.38
TTV (cm^3)	17.74	1.53	9.89	6.23	37.23	11.35	15.56	20.54	271.69	12.21	133.21	72.07	581.54	170.81	249.45	341.58
GSI (%)	0.11	0.01	0.06	0.04	0.22	0.07	0.10	0.11	0.95	0.04	0.39	0.30	1.86	0.69	0.91	1.20
Caliper Lenght LT (cm)	3.60	0.04	0.24	3.30	4.00	3.50	3.50	3.70	7.45	0.29	1.09	6.40	8.50	6.40	7.45	8.50
Caliper Lenght RT (cm)	3.64	0.09	0.55	3.00	4.50	3.20	3.50	4.00	7.80	0.33	1.25	6.60	9.00	6.60	7.80	9.00
Caliper Height LT (cm)	2.58	0.07	0.40	2.20	3.30	2.30	2.40	2.70	4.50	0.00	0.00	4.50	4.50	4.50	4.50	4.50
Caliper Height RT (cm)	2.46	0.09	0.52	2.00	3.40	2.00	2.40	2.50	4.55	0.01	0.05	4.50	4.60	4.50	4.55	4.60
Caliper Width LT (cm)	2.42	0.08	0.47	1.90	3.20	2.00	2.50	2.50	5.25	0.21	0.78	4.50	6.00	4.50	5.25	6.00
Caliper Width RT (cm)	2.30	0.07	0.41	1.90	3.00	2.00	2.10	2.50	5.65	0.24	0.88	4.80	6.50	4.80	5.65	6.50
Caliper Volume RT (cm^3)	12.19	0.80	4.74	7.94	20.45	8.35	10.07	14.13	93.96	7.25	27.13	67.81	120.10	67.81	93.96	120.10
Caliper Volume LT (cm^3)	11.59	1.09	6.47	6.59	24.02	7.33	9.54	10.46	107.01	8.53	31.92	76.25	137.76	76.25	107.01	137.76
Caliper TTV (cm^3)	23.78	1.82	10.78	14.53	44.47	17.89	20.53	21.46	200.96	15.78	59.05	144.06	257.86	144.06	200.96	257.86
Caliper GSI (%)	0.14	0.01	0.06	0.10	0.26	0.10	0.11	0.14	0.87	0.05	0.19	0.69	1.05	0.69	0.87	1.05

LT—left testicle; RT—right testicle; TTV—total testicular volume; BW—body weight; TSN—total sperm number; GSI—gonadosomatic ratio; TMS—total motile sperm count.

Table S7. 1. Posterior distribution statistics for US and caliper testicular measurements in juvenile (n=6) and mature donkeys (n=17).

**Juvenile**	**Posterior**		**95% Credible Interval**		**Mature**	Posterior		95% Credible Interval	
**Parameter**	**Mean**	**Variance**	**Lower Bound**	Upper Bound	Parameter	Mean	Variance	Lower Bound	Upper Bound
Age	11.17	62.39	-4.34	26.67	Age	79.94	22.02	70.73	89.15
Weight	160.33	56.80	145.54	175.13	Weight	279.47	20.05	270.68	288.26
Lenght LT (cm)	3.31	0.03	2.98	3.64	Lenght LT (cm)	8.23	0.01	8.03	8.42
Lenght RT (cm)	3.10	0.03	2.73	3.46	Lenght RT (cm)	8.12	0.01	7.91	8.34
Height LT (cm)	2.04	0.02	1.76	2.32	Height LT (cm)	4.90	0.01	4.73	5.07
Height RT (cm)	2.00	0.02	1.72	2.27	Height RT (cm)	4.65	0.01	4.49	4.82
Width LT (cm)	2.36	0.03	2.03	2.68	Width LT (cm)	6.17	0.01	5.98	6.36
Width RT (cm)	2.45	0.03	2.11	2.80	Width RT (cm)	6.13	0.01	5.93	6.34
Volume LT (cm^3)	9.10	86.13	-9.12	27.31	Volume LT (cm^3)	141.02	30.40	130.19	151.84
Volume RT (cm^3)	8.79	76.93	-8.42	26.01	Volume RT (cm^3)	130.68	27.15	120.45	140.91
TTV (cm^3)	17.74	318.17	-17.26	52.75	TTV (cm^3)	271.69	112.29	250.89	292.49
GSI (%)	0.11	0.00	0.00	0.21	GSI (%)	0.95	0.00	0.89	1.01
Caliper Lenght LT (cm)	3.60	0.01	3.39	3.81	Caliper Lenght LT (cm)	7.45	0.03	7.12	7.78
Caliper Lenght RT (cm)	3.64	0.02	3.37	3.91	Caliper Lenght RT (cm)	7.80	0.05	7.37	8.23
Caliper Height LT (cm)	2.58	0.00	2.46	2.70	Caliper Height LT (cm)	4.50	0.01	4.32	4.68
Caliper Height RT (cm)	2.46	0.01	2.31	2.61	Caliper Height RT (cm)	4.55	0.02	4.31	4.79
Caliper Width LT (cm)	2.42	0.01	2.23	2.61	Caliper Width LT (cm)	5.25	0.02	4.94	5.56
Caliper Width RT (cm)	2.30	0.01	2.10	2.50	Caliper Width RT (cm)	5.65	0.03	5.34	5.96
Caliper Volume RT (cm^3)	12.19	6.56	7.15	17.23	Caliper Volume RT (cm^3)	93.96	16.41	85.98	101.93
Caliper Volume LT (cm^3)	11.59	9.31	5.58	17.59	Caliper Volume LT (cm^3)	107.01	23.28	97.51	116.50
Caliper TTV (cm^3)	23.78	31.29	12.77	34.79	Caliper TTV (cm^3)	200.96	78.22	183.55	218.37
Caliper GSI (%)	0.14	0.00	0.10	0.18	Caliper GSI (%)	0.87	0.00	0.81	0.93

Table S7. 2. Summary of Bayesian ANOVA outputs to test for differences in the mean for US and caliper testicular measurements between juvenile (n=6) and mature donkeys (n=17).

Items	BG Sum of Squares	BG df	BG Mean Square	WG Sum of Squares	WG df	WG Mean Square	F	Sig.	Bayes Factor
Age	146833.58	1.00	146833.58	411416.42	159.00	2587.53	56.75	0.00	1865664731.89
Weight	440621.37	1.00	440621.37	187.04	159.00	374566.98	2355.77	0.00	30439872498826000000000000.00
Lenght LT (cm)	749.84	1.00	749.84	186.75	159.00	1.18	638.42	0.00	134300000000000000000000000000000000000000000000000000000.00
Lenght RT (cm)	784.60	1.00	784.60	226.15	159.00	1.42	551.63	0.00	1494000000000000000000000000000000000000000000000000.00
Height LT (cm)	254.24	1.00	254.24	137.93	159.00	0.87	293.07	0.00	45129474766051400000000000000000000.00
Height RT (cm)	218.76	1.00	218.76	129.47	159.00	0.81	268.67	0.00	563046624912112000000000000000000.00
Width LT (cm)	451.84	1.00	451.84	178.33	159.00	1.12	402.87	0.00	1304000000000000000000000000000000000000000.00
Width RT (cm)	420.45	1.00	420.45	200.25	159.00	1.26	333.84	0.00	41480000000000000000000000000000000000.00
Volume LT (cm^3)	540243.72	1.00	540243.72	567917.96	159.00	3571.81	151.25	0.00	5462752114363200000000.00
Volume RT (cm^3)	461201.24	1.00	461201.24	507300.12	159.00	3190.57	144.55	0.00	973169772467385000000.00
TTV (cm^3)	2001950.79	1.00	2001950.79	2097979.67	159.00	13194.84	151.72	0.00	6157121718466020000000.00
GSI (%)	22.08	1.00	22.08	18.42	159.00	0.12	190.60	0.00	68339974725802200000000000.00
Caliper Lenght LT (cm)	148.23	1.00	148.23	17.40	47.00	0.37	400.49	0.00	3905804789681000000000.00
Caliper Lenght RT (cm)	173.06	1.00	173.06	30.60	47.00	0.65	265.77	0.00	1029696219393910000.00
Caliper Height LT (cm)	36.86	1.00	36.86	5.52	47.00	0.12	314.11	0.00	28094565907231400000.00
Caliper Height RT (cm)	43.68	1.00	43.68	9.22	47.00	0.20	222.69	0.00	34033358203901200.00
Caliper Width LT (cm)	80.09	1.00	80.09	15.35	47.00	0.33	245.21	0.00	215357690762870000.00
Caliper Width RT (cm)	112.23	1.00	112.23	15.86	47.00	0.34	332.68	0.00	89059478521043600000.00
Caliper Volume RT (cm^3)	66858.42	1.00	66858.42	10334.91	47.00	219.89	304.05	0.00	14671267730557800000.00
Caliper Volume LT (cm^3)	91044.04	1.00	91044.04	14664.10	47.00	312.00	291.81	0.00	6481622507867600000.00
Caliper TTV (cm^3)	313941.699	1	313941.699	49276.47	47.00	1048.44	299.44	0.00	10821467034537700000.00
Caliper GSI (%)	5.27	1.00	5.27	0.59	47.00	0.01	416.69	0.00	8850129140121850000000.00

BG: Between groups; WG: Within groups.

Table S8. Sperm morphological abnormalities of the 40 donkey ejaculates.

Donkey	Normal morphological sperm (%)	Sperm morphological abnormalities (%)				Total abnormalities (%)
		Head	Neck	Intermediary piece	Tail	
**1**	**66.67**	0.00	0.67	0.67	32.00	33.33
2	95.50	0.00	1.00	0.00	3.50	4.50
3	93.43	1.29	0.71	4.29	0.29	6.57
4	91.93	0.67	0.00	6.24	1.17	8.07
5	92.14	0.86	1.00	3.86	2.14	7.86
6	69.43	2.43	0.43	25.57	2.14	30.57
7	86.71	0.86	1.00	8.14	3.29	13.29
8	88.50	1.33	0.33	6.50	3.33	11.50
